# Decreased flow-mediated dilation in healthy Chinese adolescent with a family history of type 2 diabetes

**DOI:** 10.1186/s12872-022-02653-2

**Published:** 2022-06-03

**Authors:** Yingwei Wang, Guangxia Li, Jing Qi, Ting Gong, Xiudong Li, Fanghe Liu, Xuejing Bi, Yang Zhao, Meihua Liang, Xiaodong Zheng, Yuandong Qiao

**Affiliations:** 1 Department of Sports Education, Harbin Medical University-Daqing, Daqing, 163319 Heilongjiang People’s Republic of China; 2 Department of Ultrosound, Longnan Hospital of Daqing, Daqing, 163453 Heilongjiang People’s Republic of China; 3 College of Pharmacy, Harbin Medical University, Harbin, 150086 Heilongjiang People’s Republic of China; 4 Department of Genetics and Cell Biology, Harbin Medical University-Daqing, 39 Xinyang Road, Gaoxin District, Daqing, 163319 Heilongjiang People’s Republic of China; 5 Clinical Laboratory, The Fifth Affiliated Hospital of Harbin Medical University, Daqing, 163319 Heilongjiang People’s Republic of China; 6 Department of Basic Nursing, Harbin Medical University-Daqing, Daqing, 163319 Heilongjiang People’s Republic of China; 7 Department of Endocrinology, The Second Affiliated Hospital of Harbin Medical University, Harbin, 150086 People’s Republic of China

**Keywords:** Flow-mediated dilation, Physical activity, Type 2 diabetes

## Abstract

**Background:**

Endothelial dysfunction appears early in the development of cardiovascular disease and is associated with type 2 diabetes. We, therefore, tested the hypothesis that endothelial dysfunction is already present in healthy Chinese adolescent participants at risk of type 2 diabetes and associates with physical activity.

**Methods:**

We investigated the flow-mediated dilation in 65 first-degree relatives (normal tension, normal glucose tolerance) and 62 age-, sex- and BMI-matched controls without a family history of type 2 diabetes by ultrasound. Physical activity level was assessed using the Global Physical Activity Questionaire and type 2 diabetes family history through self-reporting. The association between physical activity and flow-mediated dilation was evaluated by Pearson correlations and multiple regressions in adolescents with or without a family history of type 2 diabetes.

**Results:**

Female adolescents display better flow-mediated dilation than males. Adolescents with a family history of type 2 diabetes had significantly impaired flow-mediated dilation than healthy controls. Among the parameter detection in the blood, the flow-mediated dilation is only positively associated with high-density lipoprotein cholesterol level, but not others. Interestingly, flow-mediated dilation is positively corrected with physical activity scores in both the male and female adolescents, while slightly impaired but not significant in adolescents with a family history of type 2 diabetes.

**Conclusion:**

Studies in adolescents are important to understand the early pathogenesis of type 2 diabetes. Findings of this investigation suggest that family history of type 2 diabetes may play a role in regulating the vascular function in Chinese adolescents. Given the impaired flow-mediated dilation in individuals with family history and the effects of physical activity in improved flow-mediated dilation, people with a family history of type 2 diabetes may need higher physical activity levels to attenuate their susceptibility to impaired flow-mediated dilation.

## Background

Type 2 diabetes (T2D) is associated with increased incidences of potential severe outcomes, including stroke, hypertension, diabetes nephropathy, diabetes foot, and diabetes retinopathy [1]. The pathophysiology mechanisms underlying T2D including genetically programmed factors and environmental influences. It has been accepted that the vulnerable population, like the adolescents and young adults with a family history of T2D, display significantly increasing incidences of T2D, especially upon aging. Prevention of the onset of T2D in such a population should be paid attention.

Vascular function is an ideal candidate biomarker in T2D patients because of the integral role of vascular biology in developing diabetes events. Reduced endothelial function is common in diabetes patients [2]. Flow-mediated dilatation (FMD) is extensively used to evaluate endothelium function of arterial [3]. Decreased FMD is triggered by a decline in nitric oxide (NO) production or its activity in T2D patients [4, 5]. It is clear that physical activity (PA) beneficial in vascular function improvement among the aging population and under disease states [6–8]. PA results in increased blood flow, which stimulated NO in endothelial cells and the maintained activity of endothelial NO synthase [5].

The Maastricht Study indicated prediabetes and T2D are associated with generalized microvascular dysfunction [9

## Methods

### Participants

A prospective cross-sectional study was conducted in the sports education department, Harbin medical university-Daqing from January 2017 until May 2018. The exclusion criteria included diagnosed diabetes, existent cardiovascular disease, arterial hypertension, peripheral vascular disease, using glucocorticoids, or any vasoactive medications. One female was excluded as diagnosis T2D. We required participants to attend a laboratory to complete vascular function testing. All assessments were performed in a temperature-controlled quiet room. We informed participants to undertake a 4-h fast before assessing and avoiding any strenuous PA for 24 h before testing. Height and weight were determined. BMI was calculated as the body weight divided by the height (kilograms/meters squared). PA level was assessed using the International Physical Activity Questionnaire (IPAQ) short form with the local language, and family history of T2D, through self-reporting. 127 participants were enrolled in this study, including 52 male and 75 female. There are 65 healthy adolescents (age: 19.92 ± 0.15 years), with a family history of T2D in one or both parents. And 62 healthy adolescents and without parental history of diabetes (age: 20.16 ± 0.15 years) served as the control group. The study was approved by the ethics review board of Harbin Medical University-Daqing (No. HMUDQ2020091501).

### Flow-mediated dilatation

We asked the participants to control their smoking, caffeine-containing fluid, and drug intake for 12 h before performing FMD. Rest for 15 min in a supine position in a room maintained at 25 °C before the measurement. Baseline brachial artery diameter was recorded. The blood flow was cuffed by inflating the blood pressure cuff wound to 50 mmHg above the systolic blood pressure around the right forearm for 5 min. The cuff was deflated to measure any changes in the brachial artery diameter. The maximum diameter of the brachial artery within 2 min after the cuff release was recorded. Peak dilation during each study was defined as the greatest percent change from resting baseline brachial artery diameter. The echocardiographic system and the probe used were the IE33 (Philips, Netherlands) and the L11-3 (Philips, Netherlands).

### Blood tests

Fasting blood was measure in the morning immediately after endothelial function testing. The blood samples were drawn by venipuncture, then were sent for laboratory testing, including high sensitivity C-reactive protein (CRP), serum glucose, total cholesterol, high-density, low-density lipoprotein cholesterol, and glycated hemoglobin.

### Statistical analysis

Data were reported as mean ± S.E.M. Continuous variables were compared using Student's t-test between groups. Pearson correlation coefficients examined the degree of association between examined variables. All statistical analyses were performed using Graphpad Prism version 6 (GraphPad Software, San Diego, CA, USA). Pearson's correlation coefficients (two-tailed) were used to describe the strength of relationships between the dependent variables (FMD and baseline artery diameter) and potential covariates like height, weight, and BMI et al.

## Results

### Descriptive data

Clinical characteristics of the study participants are shown in Table 1. 127 participants were enrolled in this study, including 52 males and 75 females. 65 adolescents constituted the group at risk of diabetes mellitus (a group with a family history of T2D), and the remaining 62 were designated into the control group. The mean age was comparable in both groups (20.16 ± 0.16 years vs. 19.91 ± 0.14 years, *P* = 0.2695). Moreover, gender was comparable between both groups. However, BMI was significantly increased in the adolescent with a family history of T2D. Metabolic profiles revealed no significant differences in fasting serum cholesterol, high-density lipoprotein cholesterol (HDL), low-density lipoprotein cholesterol (LDL), serum glucose, high sensitivity C-reactive protein (CRP), and glycated hemoglobin (GHb), and glycated hemoglobin (GHb) levels. While serum levels of high sensitivity c reaction protein (CRP) level were elevated in those at risk of T2D but did not reach a statistical significance. (1.26 ± 0.18 vs. 1.08 ± 0.15, *P* = 0.2577) (Table 1).

**Table 1:** Demographic characteristics of Chinese adolescent with a family history versus control adolescent

	Control group (n = 62)	Group with family History of T2D (n = 65)	*P* value
Age (years)	20.16 ± 0.16	19.92 ± 0.15	0.2695
Gender (males %)	29 (46.77)	23 (35.38)	0.1920
BMI (kg/m^2^)	21.21 ± 0.33	22.57 ± 0.51	0.0300*
Vessel size (mm)	2.958 ± 0.062	2.902 ± 0.052	0.4915
Total cholesterol (mg/dL)	4.31 ± 0.09	4.35 ± 0.11	0.7487
HDL-c (mg/dL)	1.37 ± 0.03	1.33 ± 0.04	0.3962
LDL-c (mg/dL)	2.61 ± 0.10	2.62 ± 0.09	0.9269
Glucose (mmol/L)	4.70 ± 0.04	4.66 ± 0.04	0.4948
GHb	5.38 ± 0.02	5.36 ± 0.03	0.6491
CRP (mg/dL)	1.08 ± 0.15	1.38 ± 0.21	0.2577

Data are means ± S.E.M. *P* values were calculated using Student's t-test analysis

Noncategorical parameters were compared by the chi-square test

*CRP* C-reactive protein, *GHb* glycated hemoglobin

*P < 0.05

### FMD in adolescents with a family history of T2D

**Fig. 1:**
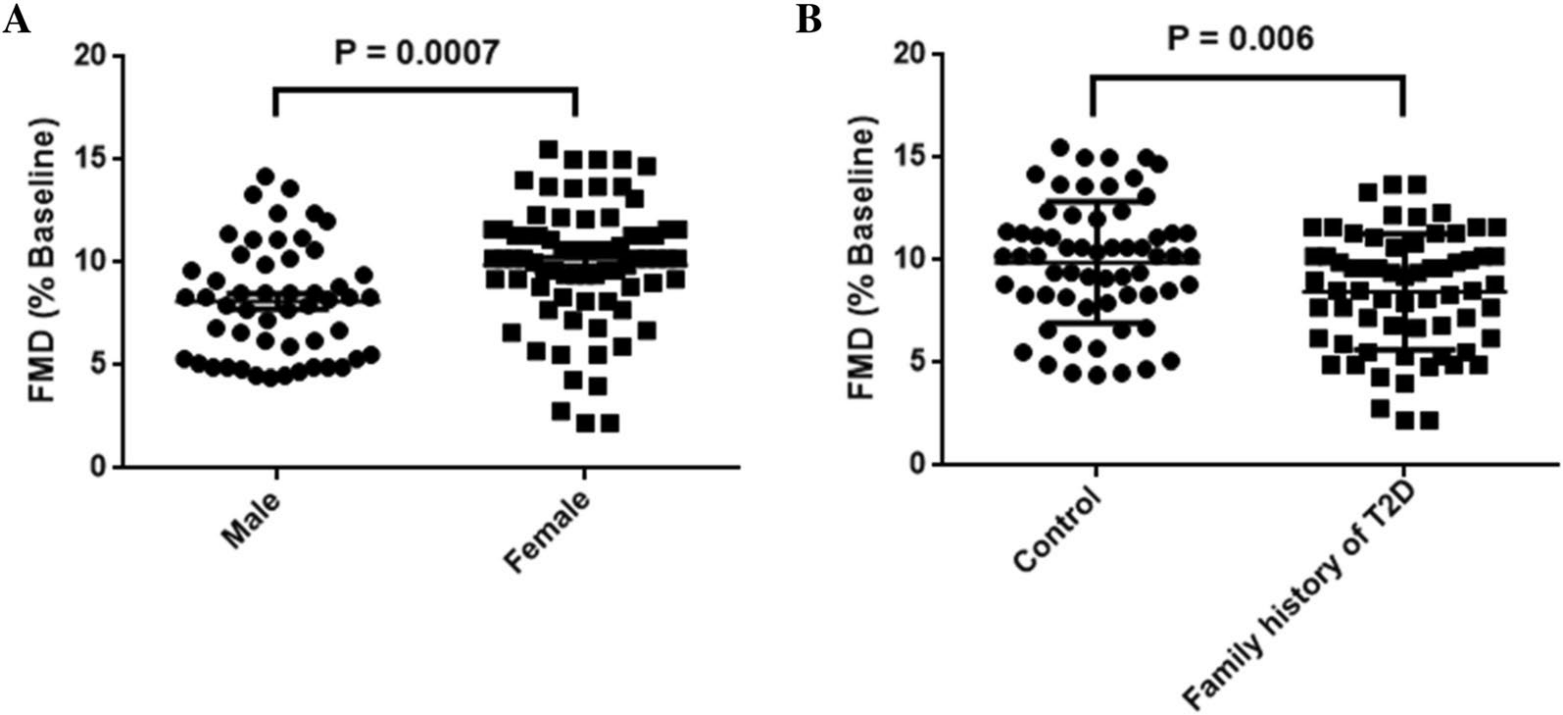
Compare FMD in control and healthy Chinese adolescents with a family history of type 2 diabetes (T2D). **A** Females display better FMD compare with the males in the whole cohort participants. **B** FMD was lower in participants with a family history of type 2 diabetes (T2D). *P* values were calculated using Student's t-test analysis

Brachial artery (BA) ultrasound analysis did not reveal any significant differences in resting diameters of both groups (2.958 ± 0.062 vs. 2.902 ± 0.052 mm, *P* = 0.4915) (Table 1). Previously study has indicated that FMD displayed the difference between male and female. In Chinese adolescent, female display better FMD compare to male (10.4 ± 0.47% vs 9.6 ± 0.52%, *P* = *0.0007*), indicated female has better vascular function (Fig. 1A). FMD was significantly decreased in adolescents with a family history of T2D compared with controls (9.07 ± 0.36% vs. 10.13 ± 0.36%, *P* = *0.006*) (Fig. 1B), the time to reach peak diameter following cuff release was not different in both group (data not shown). We also compared the FMD in adolescents with and without a family history of T2D in males and females. The results indicated that FMD is lower in male and female adolescents with a family history of T2D but did not reach statistical significance (data not shown).

### Association of FMD with metabolic profiles

We performed a stepwise multiple logistic regression analysis to identify variables that independently and significantly contributed to endothelial dysfunction detected by FMD in control and with a family history of T2D (Table 2). In a univariate analysis, analysis of the correction of FMD with total cholesterol, LDL-c, HDL-c, glucose, CRP, and GHb in control adolescent and with a family history of T2D. FMD was associated with HDL in both the control (r = 0.4619, *P* = 0.0001) and with a family history of T2D (r = 0.4960, *P* < 0.0001) adolescent. However, there was no regression interaction between the other metabolic profiles and FMD (Table 2).

**Table 2:** Associaton between metabolic profiles level and presence of FMD across the family history of T2D

	Control		With a family history of T2D	
	r (%95 CI)	*P*	r (%95 CI)	*P*
Cholesterol	− 0.1204 (− 0.3576 to 0.1313)	0.3471	0.1012 (− 0.1445 to 0.3350)	0.4190
HDL	0.4619 (0.2417–0.6368)	0.0001	0.4960 (0.2885 to 0.6589)	< 0.0001
LDL	− 0.2431 (− 0.4630 to 0.00503)	0.0549	0.08992 (− 0.1556 to 0.3249)	0.4727
Glucose	− 0.06423 (− 0.3072 to 0.1866)	0.6170	− 0.1013 (− 0.3352 to 0.1443)	0.4181
CRP	0.1207 (− 0.1310 to 0.3579)	0.3459	− 0.06875 (− 0.3058 to 0.1763)	0.5833
HbAlc	0.1074 (− 0.1442 to 0.3460)	0.4020	0.01497 (− 0.2279 to 0.2561)	0.9050

Data are presented as means ± S.E.M. or as median (interquartile range) for non-normally distributed variables. *P* values were calculated using ANOVA

*CI* confidence interval

### Association of FMD with PA

**Fig. 2:**
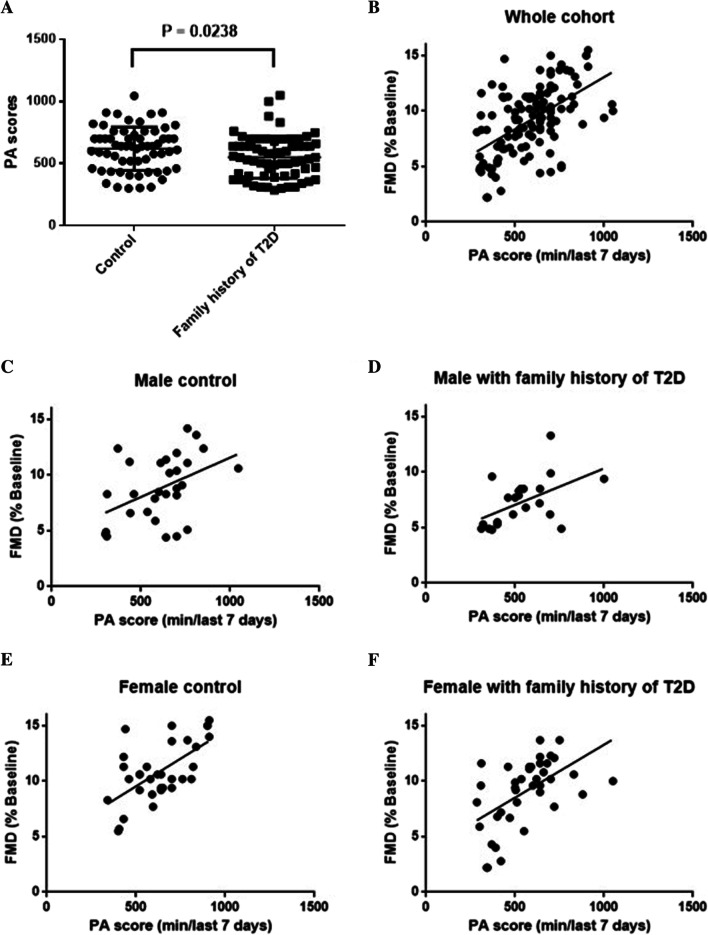
The intercorrelation between FMD and PA score. **A** Compare PA scores in control and healthy Chinese adolescents with a family history of type 2 diabetes (T2D). **B** The intercorrelation between FMD and PA scores in the whole cohort. **C** The intercorrelation between FMD and PA scores in control male adolescent. **D** The intercorrelation between FMD and PA scores in male adolescent with a family history of T2D. **E** The intercorrelation between FMD and PA scores in control female adolescent. **F** The intercorrelation between FMD and PA scores in female adolescents with a family history of T2D. *P* values were calculated using Student's t-test analysis. Correlation analysis was done by Pearson correlation

PA has been shown to benefit in the improvement of vascular function. To test the implication of PA in Chinese adolescents with and without a family history of T2D, we analyze the intercorrelation between FMD and PA. The International Physical Activity Questionnaire (IPAQ) is a comparable and standardized self-report measure of habitual PA of populations from different countries and socio-cultural contexts. There was no difference in PA scores of the last seven days between the control subject and adolescents with a family history of T2D (Fig. 2A). FMD increased with PA score (r = 0.5505, *P* < 0.0001) in the whole cohort (Fig. 2B). Interestingly, the intercorrelation increased in male adolescent with family history of T2D (r = 0.5237, *P* = 0.0103) compare with male control (r = 0.4395, *P* = 0.0170) (Fig. 2C, D), while the intercorrelation decreased in female adolescent with family history of T2D (0.5462, *P* = 0.0002) compared with female control (r = 0.6234, *P* = 0.0001) (Fig. 2E, F). These data indicated PA is beneficial in improving endothelial function, especially in the female adolescent subject with a family history of T2D.

## Discussion

The aims of this cross-sectional study including the following: (1) to determine the relationship between artery endothelial function and T2D family history in healthy Chinese adolescents, (2) to assess the relationship between serum metabolic profiles and FMD in control and with a family history of T2D, and (3) to confirm the intercorrelation between PA and FMD in healthy adolescents. The results from our study indicate that FMD was significantly decreased in adolescents with a family history of T2D and that FMD is better in females than males. Moreover, the FMD is only positively associated with high-density lipoprotein cholesterol level, but not other metabolic profiles. Furthermore, FMD was significantly correlated with PA in the whole cohort of Chinese adolescents. The intercorrelation appears to increase in a female subject. Implying PA is more important for endothelial function in participants with a family history of T2D.

The vascular endothelium plays a vital role in regulating the vascular tone [10]. Endothelial dysfunction is an early manifestation and contributes to the increased risk of development in T2D [11]. Healthy subjects with a family history of T2D display a higher risk of cardiovascular events, including T2D, especially during aging. A previous review proposed that FMD may not alter during healthy childhood and adolescence [12]. In the current study, FMD was impaired in healthy adolescent subjects with a family history of T2D, implying that T2D has its origins in childhood. The finding is controversial with a previous review that concluded that FMD was not altered during healthy childhood and adolescence [12], derived from multiple laboratory data using a different protocol. The consistency of methodology is essential for the validity and reliability of FMD. An explanation for our findings is most likely related to the “ticking clock hypothesis”, which indicated that significant artery dysfunction already occurs in prediabetes [9]. Given the growing relevance of endothelial dysfunction in this group of adolescents, a call for early preventive actions aiming to prevent the silent progression of FMD should be instated since adolescence. The activities could be including low HDL cholesterol, high LDL cholesterol, screening for overweight, fasting plasma glucose, and glycated hemoglobin. Although a family history of T2D did not affect these metabolic profiles in adolescents (Table 1), FMD declined with glucose, LDL, and BMI (Fig. 2). The small samples of the participants or the overt metabolic disease that become apparent in mid to late life could be the reasons for no statistical significance.

It is well accepted that exercise interventions can improve endothelial function [13, 14]. Our data showed that FMD was significantly related to PA in the whole cohort. The correlation between FMD and PA appears to be more significant in subjects with a family history of T2D. It indicated that PA could serve as an essential preventive factor for endothelial dysfunction. It was supported by that high-intensity interval training leads to a superior reduction in night-time diastolic BP [15], improvement of cardiorespiratory fitness, some inflammatory markers, and anxiety and depression [16].

Some key limitations should be taken into account in the current study. First, our FMD data was not adjusted with the brachial artery diameter. Since the diameter displayed significant associations with age and sex, females have a smaller diameter than males, explaining the better FMD. Moreover, brachial artery diameter increased with age. Due to insufficient numbers of adolescents with the necessary year stages, we cannot definitively comment on the age-related effects on FMD. Second, due to the systemic natures (interval exercise, various neurogenic, hormonal et al.), which may affect the FMD, the underlying factors modulating the endothelium-dependent vasodilation remain unclear. It has been suggested that different modes of exercise and training status can modify endothelial shear stress and result in distinct effects on endothelial function in T2D patients [7]. FMD is diminished by reducing extracellular calcium and sodium [17] and is improved by magnesium [18]. Due to the various modes of exercise involved in our study, and the ion concentration was not designed to measure, we cannot assess which kinds of exercise were suitable and the implication of serum ion concentration for FMD in adolescents with a family history of T2D.

## Conclusion

This study aimed to empirically measure the relationship between FMD and the family history of T2D in healthy Chinese adolescents. Our analysis indicates that there appear to be differences in FMD between control and family history of T2D adolescents. Additionally, we demonstrate a correlation between PA and FMD, especially in participants with a family history of T2D, suggesting that sustaining PA may improve the endothelial function in Chinese adolescents.

## Data Availability

The datasets used and/or analysed during the current study are available from the corresponding author on reasonable request.

## Abbreviations

T2D: Type 2 diabetes
PA: Physical activity
FMD: Flow-mediated dilation
NO: Nitric oxide
IPAQ: International physical activity questionnaire
